# Segmentation of pulmonary nodules using adaptive local region energy with probability density function-based similarity distance and multi-features clustering

**DOI:** 10.1186/s12938-016-0164-3

**Published:** 2016-05-05

**Authors:** Bin Li, QingLin Chen, Guangming Peng, Yuanxing Guo, Kan Chen, LianFang Tian, Shanxing Ou, Lifei Wang

**Affiliations:** School of Automation Science and Engineering, South China University of Technology, Guangzhou, 510640 Guangdong China; Department of Radiology, Guangzhou General Hospital of Guangzhou Command, Guangzhou, 510010 Guangdong China; Department of Radiology, Shenzhen Third People’s Hospital, Shenzhen, 518112 Guangdong China

**Keywords:** Image segmentation, Pulmonary nodules, Active contour model, Feature extraction, Computer tomography image

## Abstract

**Background:**

Pulmonary nodules in computerized tomography (CT) images are potential manifestations of lung cancer. Segmentation of potential nodule objects is the first necessary and crucial step in computer-aided detection system of pulmonary nodules. The segmentation of various types of nodules, especially for ground-glass opacity (GGO) nodules and juxta-vascular nodules, present various challenges. The nodule with GGO characteristic possesses typical intensity inhomogeneity and weak edges, which is difficult to define the boundary; the juxta-vascular nodule is connected to a vessel, and they have very similar intensities. Traditional segmentation methods may result in the problems of boundary leakage and a small volume over-segmentation. This paper deals with the above mentioned problems.

**Methods:**

A novel segmentation method for pulmonary nodules is proposed, which uses an adaptive local region energy model with probability density function (PDF)-based similarity distance and multi-features dynamic clustering refinement method. Our approach has several novel aspects: (1) in the proposed adaptive local region energy model, the local domain for local energy model is selected adaptively based on k-nearest-neighbour (KNN) estimate method, and measurable distances between probability density functions of multi-dimension features with high class separability are used to build the cost function. (2) A multi-features dynamic clustering method is used for the segmentation refinement of juxta-vascular nodules, which is based on the nodule segmentation using active contour model (ACM) with adaptive local region energy and vessel segmentation using flow direction feature (FDF)-based region growing method. (3) it handles various types of nodules under a united framework.

**Results:**

The proposed method has been validated on a clinical dataset of 113 chest CT scans that contain 157 nodules determined by a ground truth reading process, and evaluating the algorithm on the provided data leads to an average Tanimoto/Jaccard error of 0.17, 0.20 and 0.24 for GGO, juxta-vascular and GGO juxta-vascular nodules, respectively.

**Conclusions:**

Experimental results show desirable performances of the proposed method. The proposed segmentation method outperforms the traditional methods.

## Background

**Fig. 1:**
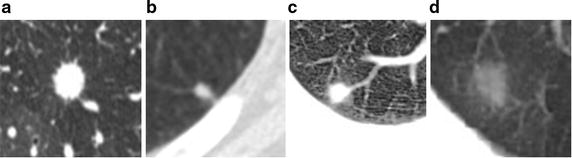
Some classical pulmonary nodules. **a** Well-circumscribed nodule; **b** Juxta-pleural nodule; **c** Juxta-vascular nodule; **d** GGO nodule

Pulmonary nodules in high resolution computerized tomography (CT) images are potential manifestations of lung cancer. As pointed by literatures [1, 2, 3, 4], despite much effort being devoted to the nodule segmentation problem, segmentation for various types of pulmonary nodules remains an ongoing research topic [4]. Some classical pulmonary nodules are shown in Fig. 1. One of the major difficults is the task of segmentation of non-solid and part-solid ground-glass opacity (GGO) nodules with faint contrast and fuzzy margins (shown as Fig. 1d). In particular, non-solid nodules are extremely subtle with fuzzy boundaries, and part-solid nodules exhibit highly irregular intensity variations (called intensity inhomogeneity) and boundary shapes. Studies have shown that nodules of non-solid and part-solid nature are frequent and have higher risks of being malignant than solid ones [1]. Additionally, there is difficulty associated with the segmentation of nodules that are adjacent to vessels when they have very similar intensities (shown as Fig. 1c Juxta-vascular nodule); and are nonspherical in shape. Juxta-vascular nodules account for the largest typology of lung nodules [2]. Thus, handling them under a united framework poses a great challenge to the task of segmentation of pulmonary nodules. Although various algorithms have been reported in literatures [1, 2, 3, 4, 5] for tackling these problems, the technical issues of segmentation still remain. Traditional segmentation methods, such as purely intensity thresholding or model-based segmentation methods, may fail to segment various types of nodules, especially GGO and juxta-vascular nodules, leading to boundary leakage or over-segmentation. All these factors lead to the belief that the field is relatively new and requires further investigation. This paper deals with the above mentioned problems. In this paper, a novel segmentation method is proposed for various types of pulmonary nodules in CT images, especially for GGO nodules (part-solid and nonsolid) and juxta-vascular nodules.

### Previous work on segmentation of pulmonary nodules

The segmentation under a united framework of kinds of pulmonary nodules, especially for GGO nodules and juxta-vascular nodules, is a very difficult task. Active contour models (ACMs) have been one of the most successful methods for image segmentation [6, 7, 8, 9, 10], even in the segmentation of pulmonary nodules [11]. However, GGO nodules possess weak edges, intensity inhomogeneity and irregular shape, so when they is segmented by using traditional edge-based ACMs [12], region-based ACMs [6, 7], or even some complex integrated ACMs combine edge and region energy [13, 14], they often occur the problem of boundary leakage. Generally, the model using local information commonly can obtain better performance than that of global statistic information in solving the segmentation problems of intensity inhomogeneity and zigzag edge [7, 15, 16, 17]. The idea of defined local neighbor is more reasonable, especially for segmenting the zigzag and inhomogeneous edge. Li et al. [7] presented a region-based active contour model (ACM) model, in which a data fitting energy is introduced to solve the problem of intensity inhomogeneity. Lankton et al. [16] summarized and proposed different segmentation models based on local information. But typically, the segmentation curve cannot obtain exact edge or deviates from the objects if the local domain is too large or too small [17]. Further, an active contour model based on nonparametric independent and identically distributed statistics of the image may segment an image according to the particular global to local strategy. The local histogram method using Wasserstein distance to measure distribution distance has a good performance in segmenting cluttered scenes [15]. However,the estimation effect in image segmentation is severely influenced by the small cabin volume and the sample distribution, when the pixel density functions are estimated by histogram method. It is difficult to acquire an ideal segmentation effect only by relying on general intensity-data-driven segmentation methods. In order to overcome the limitation, Krinidis et al. [18] used fuzzy energy to solve the problem of “weak” local minima. Also Assen et al. [19] presented a 3D ACM drived by fuzzy inference for cardiac CT and MR images. Zhang et al. [20] added the Bayesian error of edge direction and region statistical information into the ACM model, to improve the convergence speed.

As mentioned above, juxta-vascular nodules account for the largest typology of lung nodules [2]. So besides handling GGO nodules with intensity inhomogeneity and weak edges, it is also important for a segmentation algorithm to be able to treat juxta-vascular nodules. In clinic application, even some nodules are not only GGO but also juxta-vascular nodules. How to handle various types of pulmonary nodules, including GGO nodules and juxta-vascular nodules, under a united framework presents a great challenge [2, 3]. Since vessels can be characterized by the tubular models and a pulmonary nodule is a small round or oval-shaped growth in the lung, so many approaches based on morphological operators [2, 21, 22] have been proposed to segment the juxta-vascular nodules. However the sizes and shapes of vessels as well as those of nodules are irregular, it may lead to the problem of a small volume overestimation if only morphological correction is relied upon. Hence a better segmentation refinement method should be taken into consideration furtherly. Besides intensity feature, the analysis of the shape of pulmonary structures has often been adopted to recognize small lung nodules from the background anatomy [2]. However, approaches utilizing simple criteria like shape rule or gray value evidence are typically not suitable to differentiate between different tubular tree structures and nodules. Lung nodules are embodied in a complex and structured background. Their identification and segmentation is usually affected by surrounding anatomical objects [2]. So, in a broad sense, the feature space for the recognition of nodules should be embed more prior information, including the target structures [23, 24].

To our knowledge, there are few literatures aimed at handing GGO and juxta-vascular nodules under a united framework and multi-features classification space. In our previous work, we have built a very preliminary fuzzy integrated ACM incorporated multi-features analysis to realize segmentation of GGO and juxta-vascular nodules [14, 25, 26]. This paper deals with the above mentioned problems further. In our present study, the segmentation problem is converted into the optimization problem of measurable distance between probability density functions of multi-features. A multi-features dynamic clustering method is used for the segmentation refinement of juxta-vascular nodules.

### Our approach

**Fig. 2:**
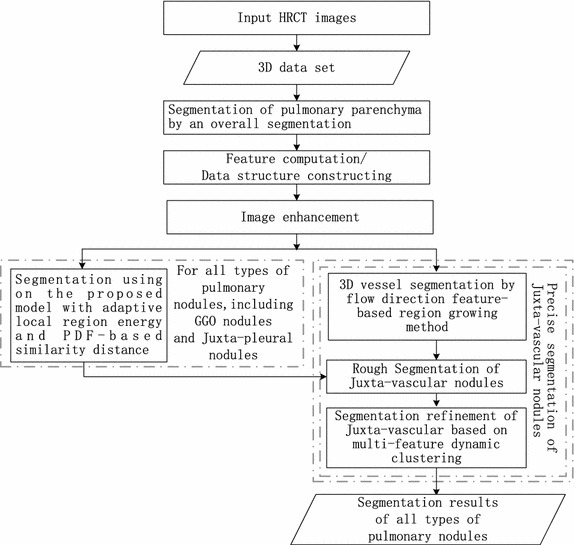
Overview of the proposed segmentation method for pulmonary nodules

In this paper, a novel segmentation method for pulmonary nodules, especially for GGO nodules and juxta-vascular nodules in CT images is proposed, which uses an adaptive local region energy model with probability density function (PDF)-based similarity distance and multi-features dynamic clustering refinement method. The flowchart of the proposed segmentation algorithm for pulmonary nodules under a united framework is shown as Fig. 2.

Compared with existing traditional methods, our approach has several novel aspects: (1) in the proposed adaptive local region energy model, the local domain for local energy model is selected adaptively based on k-nearest-neighbour (KNN) estimate method, and measurable distances between probability density functions of multi-dimension features with high class separability are used to build the cost function. (2) A multi-features dynamic clustering method is used for the segmentation refinement of juxta-vascular nodules, which is based on the nodule segmentation using active contour model with adaptive local region energy and vessel segmentation using flow direction feature (FDF)-based region growing method. (3) It handles various types of nodules under a united framework.

The remainder of this paper is organized as follows. In “The proposed integrated ACM with adaptive local region energy and PDF-based similarity distance” section and “Segmentation refinement of juxta-vascular nodules based on multi-features dynamic clustering” section, the proposed segmentation methods of pulmonary nodules are introduced. The experimental results of our method are given in “Experimental results” section, followed by some discussions in “Discussion” section. This paper is summarized in “Conclusions” section.

## The proposed integrated ACM with adaptive local region energy and PDF-based similarity distance

We will approach the segmentation problem as a typical optimization task. Thus we need to adopt an appropriate model of energy function as the cost function. The proposed integrated ACM with adaptive local region energy and PDF-based similarity distance differs from the model used in literatures [7, 13, 14, 18, 19], which has the following characteristics.

1. The KNN-based adaptive local energy function model is proposed. The nodule with GGO characteristic is either part-solid or nonsolid, in which case it possesses typical weak edges and intensity inhomogeneity. The model using local information commonly can obtain better performance than that of global statistic information in solving the segmentation problem [7, 15, 16]. The local domain for local energy model is selected adaptively, which is approached as nonsupervised recognition problem and realized by a KNN estimate method based on medical prior knowledge. It will be explained in detail in “The proposed KNN-based adaptive local energy function” section.
2. The segmentation problem is converted into the optimization problem of measurable distance between PDF of multi-features. The Bhattacharyya distance function is applied to measure the distance of PDF between the foreground and background, and the PDF in the local region are measured by Wasserstein distance. It will be explained in detail in “The similarity distance based on adaptive local region probability density” section and “The PDF-based Bhattacharyya similarity distance for global energy” section.
3. Multi-features information with high class separability is reflected and used in the proposed integrated ACM model. It will be explained in detail in “Generation of multi-dimension feature with high class separability” section.

Taking both the edge, local and global region information into consideration, our proposed energy function of integrated active contour model *E* is given as Eq. (1).

1 $$ E = E_{edge} + E_{local} + E_{global}$$

where *E* is the proposed energy function model; *E*_*edge*_ it the edge-driven energy term, which is used for curved surface to improve the evolved ability in concave regions; *E*_*local*_ is the local-region-driven energy term; *E*_*global*_ is the global-region-driven energy term. *E*_*local*_ and *E*_*global*_ are used to control the image force based on statistical multi-features information in the region and move the curved surface in the decrescence direction of feature variance.

Let $\Upomega \subset R^{ 3}$ be the image domain, and $D:\Upomega \to R$ be the given medical CT image sequence or 3D data set. The segmentation result of the images or data set (for 3D data set) *D* is achieved by finding a surface $\phi$, which separates Ω into disjoint regions. Ω_1_ and Ω_2_ represent the inside regions and outside regions of $\phi$, respectively. Besides intensity, more features are used in our active contour model. Our proposed detailed energy function model is given as Eq. (2) based on Eq. (1). Why and how to build the energy term model as Eq. (2) will be explained in detail in the following sections (from “The proposed KNN-based adaptive local energy function”, “The similarity distance based on adaptive local region probability density”, “Generation of multi-dimension feature with high class separability”, “The PDF-based Bhattacharyya similarity distance for global energy” section).

2 $$ \left\{ \begin{aligned} &E(\phi ) = E_{edge} + E_{local} + E_{global} \\ &E_{edge} = \mu \int_{\phi } {\delta \phi (v)g|\nabla \phi } |dv\\ &E_{local} = \int_{{\Upomega_{ + } }} {\lambda [1 - u(v)]^{m} } H(\phi (v))\int_{{L_{\tau } }} {\left\| {P(v) - P_{1} (\tau )} \right\|} d\tau )dv \\ &\quad\quad\quad + \int_{{\Upomega_{ - } }} {\lambda_{2} [1 - u(v)]^{m} } (1 - H(\phi (v)))\int_{{L_{\tau } }} {\left\| {P(v) - P_{1} (\tau )} \right\|} d\tau )dv \\ &E_{global} = \int_{\Upomega } {\sqrt {P_{ - } (\tau )P_{ + } (\tau )d\tau } }  \end{aligned} \right. $$

where *E*($\phi$) is the proposed energy function model; $v\left( {x, y, z} \right) \in \,\Upomega$ is a given pixel/voxel. In term *E*_*local*_ of Eq. (2), *λ*_1_ and *λ*_2_ are the weights for region-driven energy term. The membership function $u\left( v \right) \, \in \,\, \left[ {0, \, 1} \right]$ is the degree of membership of *D*, and *m* is a weighting exponent on each fuzzy membership. The degree of membership is decided by multi-features value *L*. *L* is the value domain of feature space. $H \phi$ is the smoothed Heaviside function. *P*(*v*) is the probability density of multi-features vector *O* of *v*; *P*_1_(*τ*) and *P*_2_(*τ*) are the means of probability density in local region Ω_*τ*_ selected adaptively. In term *E*_*global*_ of Eq. (2), *P*_−_(*τ*) and *P*_+_(*τ*) are the kernel-based estimates of the image features observed over the sub-domains Ω_−_ and Ω_+_. *E*_*edge*_ is similar to our previous work [14], which is used for curved surface to improve the evolved ability in concave regions. In term *E*_*edge*_ of Eq. (2), *μ* is the weight of edge-driven energy term; *δ*$\phi$(*v*) is the smoothed version of the Dirac delta; *g* is the stop function.

### The proposed KNN-based adaptive local energy function

The model using local information commonly can obtain better performance. The proposed local energy function model is inspired initially by literature [7, 15]. But in our proposed local energy function model, the local domain is not fixed, but flexible and adaptive, which is realized by a KNN estimate method here. In order to segment elaborately for the image with zigzag edge and noise interference, we construct k-nearest neighbors and estimate the corresponding probability density functions with Parzen window method in each pixel/voxel.

**Fig. 3:**
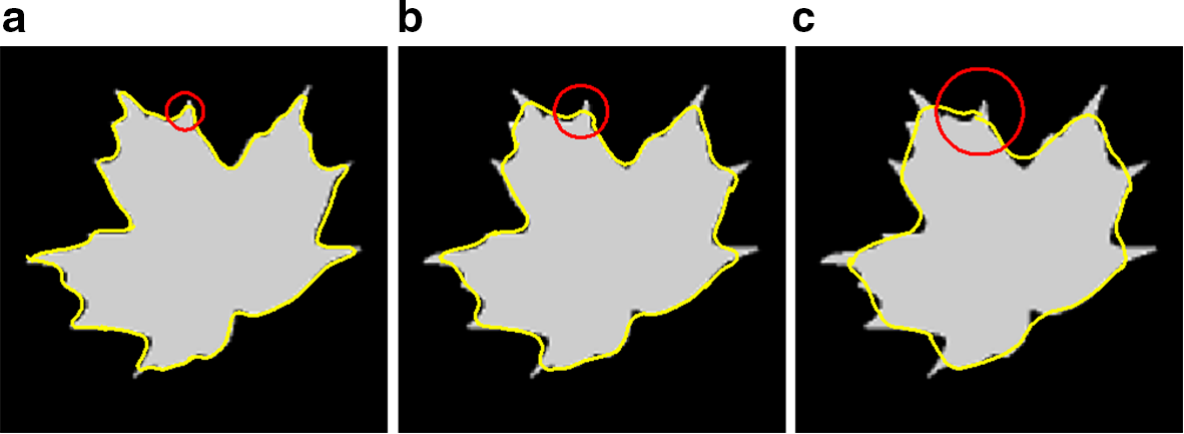
The segmentations with different neighbor radii. The radii of **a**–**c** are 3, 10, 15

As we have known, lung nodules, especially malignant nodules, sometimes show spiculation and lobulation signs in CT images. Typically, the segmentation curve deviates from the objects if the local neighbor radius is too large or too small. Consider the synthetic image in Fig. 3. The segmentation results are very different with various radii with the implementation of local histogram-based segmentation method using the Wasserstein Distance [15]. For instance, conventional local segmentation algorithms often suffer from disturbances induced by the mass of irrelevant information when the radius of the neighbor increases. To improve the ability to segment precisely the object where is intensity inhomogeneity, zigzag and difficult to define the boundary, the local region Ω_*τ*_ of $\int_{{L_{\tau } }} {\left\| {P(v) - P_{1} } \right\|} d\tau$ and $\int_{{L_{\tau } }} {\left\| {P(v) - P_{2} } \right\|} d\tau$ in term *E*_*local*_ of Eq. (2), should be selected adaptively. So the KNN-based adaptive local energy function model is proposed. The local domain for local energy model is selected adaptively, which is approached as nonsupervised recognition problem and realized by a KNN estimate method based on medical prior knowledge.

Let x, y, z be the location variables of *v*, respectively. For each current voxel *v*, selecting a circular (sphere) neighbor domain *R*_*L*_ with radius *r*; selecting *k* nearest neighbors of *O*(*v*) to construct the local domain *N*_*k*_, we have the complementary set *N*_*c*_ of *N*_*k*_ in the *R*_*L*_. If each voxel of *R*_*L*_ is met the condition as Eq. (3), select *R*_*L*_ as the local region for computing similarity in Eq. (3).

3 $$ d(O({\text{x), }}O ( {\text{y))}} \le d(O({\text{x), }}O ( {\text{z))}} $$

where ${\text{y}} \in N_{k} ,{\text{ z}} \in N_{c} ;\,\,d\left( {O\left( {\text{x}} \right), {\text{O}}\left( {\text{y}} \right)} \right)$ is the similarity distance, as Eq. (4).

4 $$ d(O({\text{x),O(y))}} = \left\| {J(O({\text{x)) }} -J ( {\text{O(y))}}} \right\| $$

where *J*(*O*) is the normalized multi-features vector *O*.

**Fig. 4:**
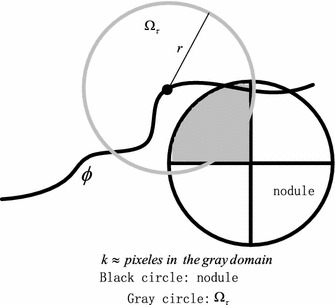
Illustration of selecting adaptively local region and parameters *r*, *k*

In the KNN-based adaptive local energy function model, the parameters *r* and *k* are determined by medical prior knowledge and the physical resolution of the pixel in CT imaging. As shown in Fig. 4, the local region Ω_*τ*_ in 2-dimensional space is determined by the parameters *r* and *k*. Rules for determining the parameters *r* and *k*, as well as their reasons, are as follows.

Rule 1: *r* and *k* are selected as large as possible
Rule 2: *r* ≤ (8 *mm*/2)/(*pixel spacing*)

Reasons are below: (1) The best choice of *k* depends upon the data. Generally, larger values of *k* reduce the effect of noise on the classification, but make boundaries between classes less distinct. (2) A pulmonary nodule is a small round or oval-shaped growth in the lung. It is sometimes also called a spot on the lung or a coin lesion. So the local region Ω_*τ*_ is selected by a circle (a sphere for 3-dimensional space). (3) $k \le \pi r^{ 2}$ (for $N_{k} \subset \,\Upomega_{\tau } ,$ in 2-dimensional space). So *r* is selected as large as possible. (4) Pulmonary nodules are generally smaller than 3 cm (about ≤3 cm) in diameter. If the growth is larger than that, it is known as a pulmonary mass. (5) The nodule may have first been identified by a CT scan. CT scans can give information about the specific features of the nodule, including its shape, size, location and internal density. A CT scan can find very small nodules, as small as 1–2 mm in diameter. Most benign (not cancerous) nodules are small (less than 5 mm) in size. Most nodules between 5 and 10 mm will need additional imaging unless they are unlikely to be cancer based on the way they look. Larger nodules require more careful evaluation and examination including additional imaging tests and possibly a biopsy. So we can determine the parameters *r* and *k* according to the above medical prior knowledge. (6) According to medical knowledge, nodules less than 8 mm are usually too small for a biopsy. In other word, the nodule more than 8 mm should be evaluated carefully. In other words, we should make sure of the distinct in the local region of the nodule which is more than 8 mm.

Rule 3: $$ {{k \, \le \,\pi ({8\,mm} /2(pixel \,\,spacing))^2}}/K_{0}, \quad K_{0}=4$$

Reasons are below: (1) The local region Ω_*τ*_ is selected by a circle (for 2-dimensional space). (2) $N_{k} \subset \,\Upomega_{\tau }$. (3) Value of *k* should make boundaries between classes more distinct in the local region. (4) The following experiment of a dataset (the phantom nodules) was performed to determine and test the parameters.

**Fig. 5:**
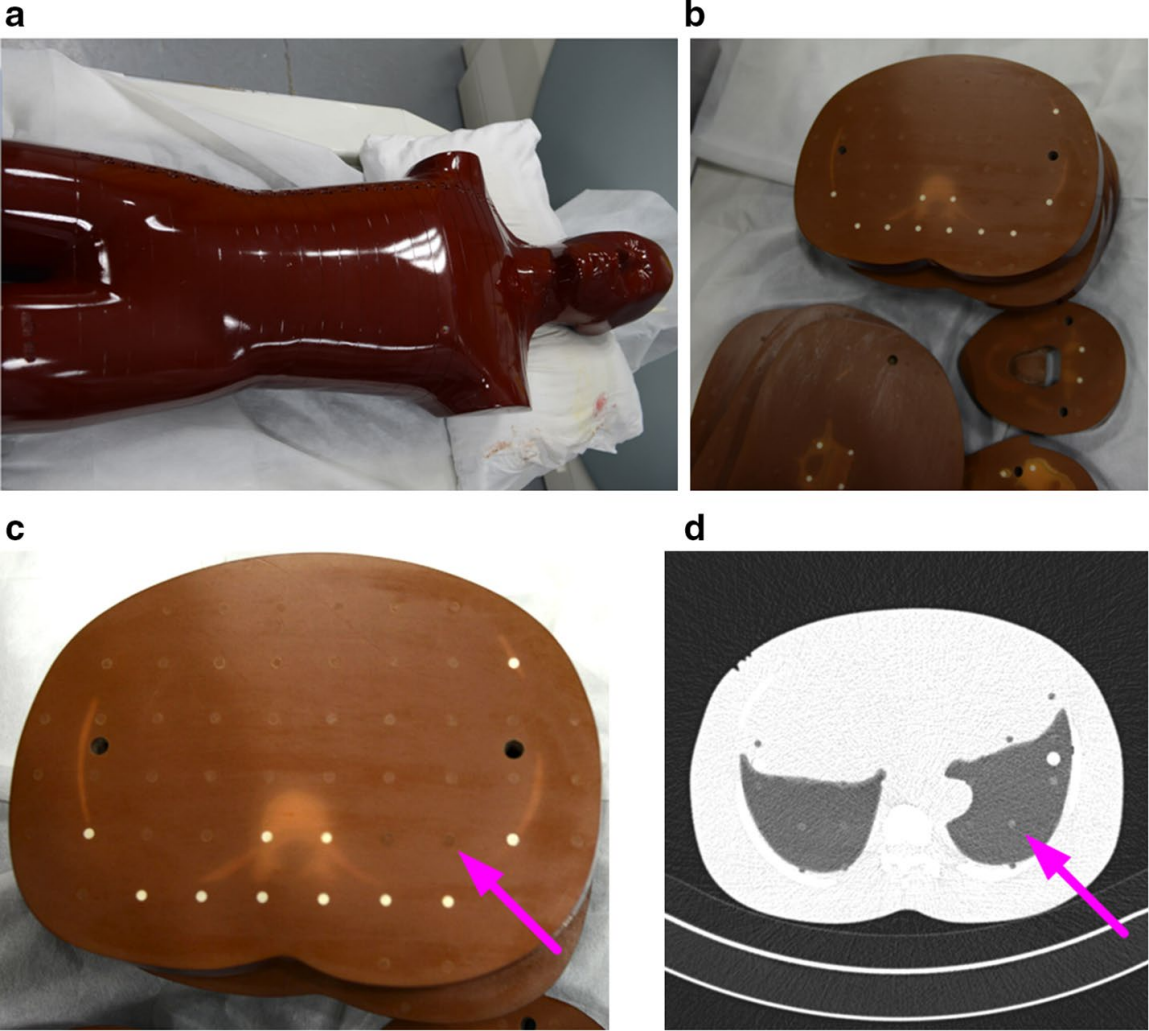
The phantom nodules. **a** The appearance of the phantom; **b** interior of the phantom; **c** nodule models in the phantom (5 mm in diameter, from −406 to −477 HU); **d** the corresponding CT image (lung from −618 to −726 HU)

In the experiment, some nodule models (5 mm in diameter, from −406 to −477 HU, shown as Fig. 5c) in a phantom (shown as Fig. 5a) are used to determine and test the parameters. A quantitative analysis was performed to determine the parameters. The well known Tanimoto/Jaccard error *A*(*C*_*m*_, *C*_*o*_) is used as the validation merics, which refers to volume overlaps between the gold standard and the proposed segmentation method with different *K*_0_. In the experiment, the gold standard is the boundary of the phantom nodule, which is shown as Fig. 5d. *A*(*C*_*m*_, *C*_*o*_) is defined as Eq. (5).

5 $$ A(C_{m} ,C_{o} ) = 1\frac{{\int_{{C_{m} \cap C_{o} }} {dxdydz} }}{{\int_{{C_{m} \cap C_{o} }} {dxdydz} }} $$

where *C*_*m*_ and *C*_*o*_ are the extracted and the desired contours, respectively.

**Fig. 6:**
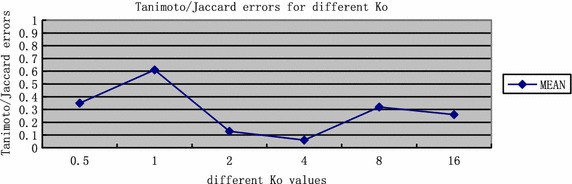
Segmentation results for different *K*_0_

Here, ${{k = \pi r^{ 2} } \mathord{\left/ {\vphantom {{k = \pi r^{ 2} } {K_{0} }}} \right. \kern-0pt} {K_{0} }}\cdot r$ is selected as 5 mm/(2 pixel spacing), which is corresponded to the size of the phantom nodule (5 mm in diameter). Segmentation results for different *k*_0_ are shown in Fig. 6. Figure 6 shows that the segmentation result with *K*_0_ = 4 is the best.

### The similarity distance based on adaptive local region probability density

The segmentation problem is converted into the optimization problem of measurable distance between probability density functions. In this paper the probability density *P*(*v*) of multi-features vector *O* for the pixel/voxel *v* is estimated by the Parzen window estimation method. Gaussian function is used as the kernel function. The probability density function of neighbor pixel is represented as Eq. (6).

6 $$ P(v) = \frac{1}{k}\int_{{N_{x} }} {K(||O(v)|| - ||O({\text{x}})||)} d{\text{x}} $$

where $K(\tau ) = (\sqrt {2\pi } \sigma )^{ - 1} \exp \{ - \tau^{2} /2\sigma^{2} \}$ is a kernel function, and generally as Gaussian density kernel function, and $\smallint K\left( \tau \right)d\tau = 1$.

The similarity distance *W*(*P*, *P*_1_) between *P*(*v*) and *P*_*i*_ is computed by Wasserstein distance function, as Eq. (7).

7 $$ W(P,P_{i} ) = \int_{{L_{\tau } }} {||P(v) - P_{i} (\tau )||} d\tau |_{i = 1,2} $$

where *L*_*τ*_ is the value domain of feature space, *P*(*v*) is the probability density of multi-features vector *O* of *v*; *P*_*i*_(*τ*) are the means of probability density in local region Ω_*τ*_ selected adaptively, which is as Eqs. (8) and (9).

8 $$ P_{1} (\tau ) = \overline{P(\tau )},  \quad {\text{where}}\, \left\{ {v:\phi \left( v \right) \ge 0} \right\} $$

9 $$ P_{2} (\tau ) = \overline{P(\tau )}, \quad{\text{where}}\,\,\left\{ {v:\phi \left( v \right) < 0} \right\} $$

### Generation of multi-dimension feature with high class separability

For one of the major difficults is the task of segmentation of non-solid and part-solid GGO nodules with faint contrast and fuzzy margins. In particular, non-solid nodules are extremely subtle with fuzzy boundaries, and part-solid nodules exhibit highly irregular intensity variations (intensity inhomogeneity) and boundary shapes. GGO pulmonary nodules are hard to distinguish if merely the intensity feature is utilized. Multi-features information with high class separability is reflected and used in the proposed integrated ACM model.

Here, in the feature space, a sample is *O*_*i*_ = (*x*_*i*_, *y*_*i*_, *z*_*i*_, *I*_*i*_, *level of ASM*_*i*_); *x*_*i*_, *y*_*i*_ and *z*_*i*_ is the position feature; *I*_*i*_ is the intensity feature and *ASM*_*i*_ is the angular second moment (texture feature) which is as Eq. (10).

10 $$ ASM = \sum\limits_{m = 0}^{{N_{g} - 1}} {\sum\limits_{n = 0}^{{N_{g} - 1}} {(P(m,n))^{2} } } $$

where *P*(*m*, *n*) is the matrix element of the normalized gray-level co-occurrence matrix. *N*_*g*_ is the number of possible gray levels. A gray level co-occurrence matrix is a matrix where the number of rows and columns is equal to the number of gray levels. The matrix element *P*(*m*, *n* | Δ*x*, Δ*y*) is the relative frequency with which two pixel, separated by a pixel distance (Δ*x*, Δ*y*), occur within a given neighborhood, one with intensity *m* and the other with intensity *n*. Given an *M* × *N* neighborhood, let *f* (*m*_*t*_, *n*_*t*_) be the intensity at sample *s*, line *l* of the neighborhood. *P*(*m*, *n* | Δ*x*, Δ*y*) is defined as Eq. (11).

11 $$ P(m,n|\Updelta x,\Updelta y) = WQ(m,n|\Updelta x,\Updelta y) $$

where

$$ W = \frac{1}{(M - \Updelta x)(N - \Updelta y)} $$

$$ Q(m,n|\Updelta x,\Updelta y) = \sum\limits_{l = 1}^{N - \Updelta y} {\sum\limits_{s = 1}^{M - \Updelta x} A } $$

and

$$ A = \left\{ {\begin{array}{*{20}ll} 1 \quad& {if\;f(s,l) = m\quad {\text{and}}\quad f(s + \Updelta x,l + \Updelta y) = n} \\ 0 \quad& {elsewhere} \\ \end{array} } \right. $$

This feature *ASM*_*i*_ is a measure of the smoothness of the image. Indeed, if all pixels are of the same gray-level *I* = *k*_I_, then *P*(*k*_I_, *k*_I_) = 1 and *P*(*m*, *n*) = 0, *m* ≠ *k*_I_ or *n* ≠ *k*_I_, and *ASM* = 1. At the other extreme, if we could have all possible pairs of gray levels with equal probability $\frac{1}{R}$, then $ASM = \frac{1}{R}$. The less smooth the region is, the more uniformly distributed *P*(*i*, *j*) and the lower the *ASM*.

**Fig. 7:**
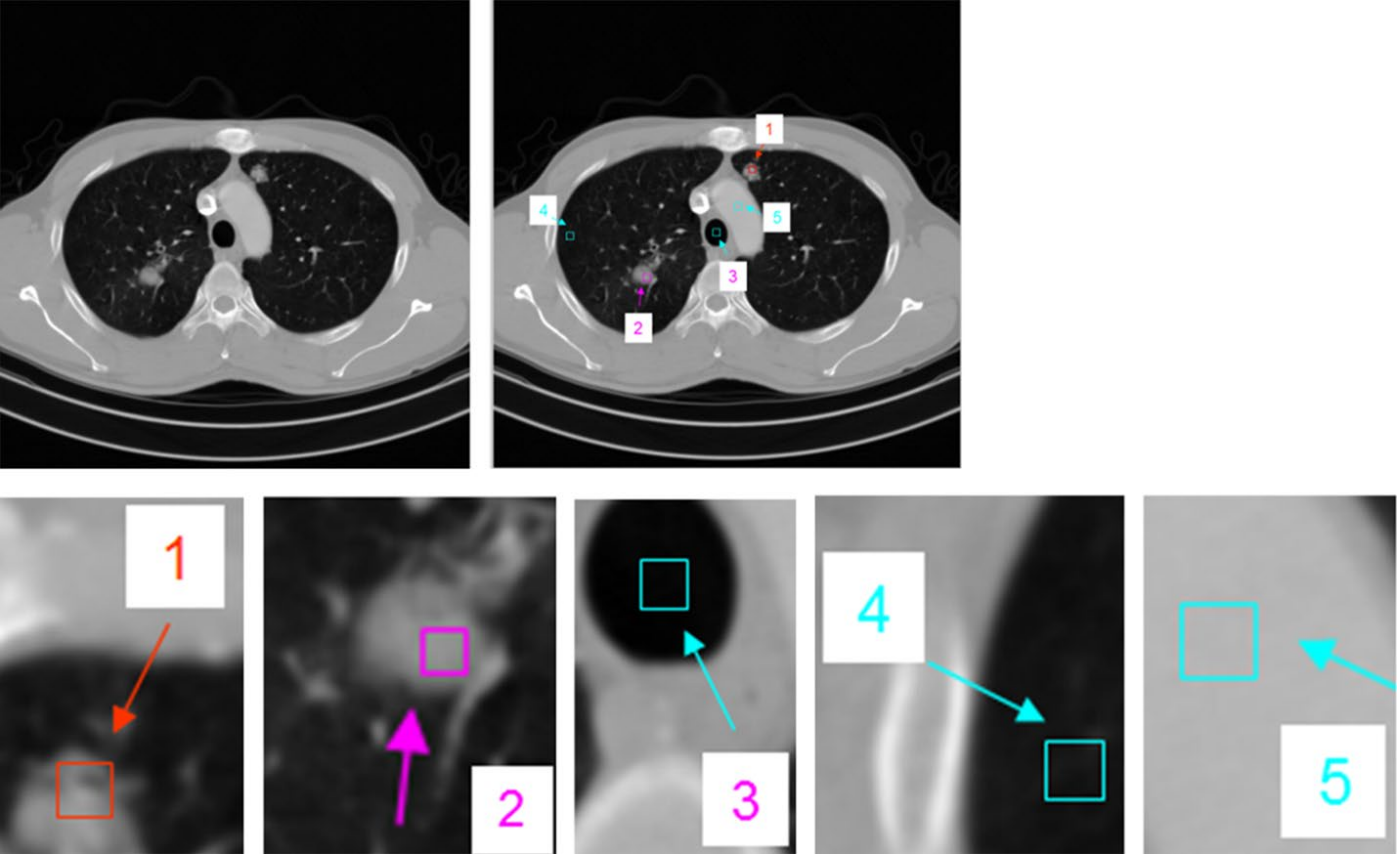
Different region (size 8×8). region 1, GGO nodule; region 2, solid nodule; region 3, vessel; region 4, parenchyma; region 5, myocardium

An example is illustrated in Fig. 7 and Table 1. It implies that the *ASM* value is an important feature for segmentation of GGO nodules, solid nodules or other regions.

**Table 1:** Second order histogram (angular second moment, ASM) feature value for different region

	1	2	3	4	5
	GGO nodule	Solid nodule	Vessel	Parenchyma	Myocardium
ASM	0.2212	0.2448	0.4828	0.4817	0.6403

The segmentation problem is converted into the optimization problem of measurable distance between PDF *P*(*v*) of multi-features *O*, shown in Eq. (2). In proposed integrated ACM model, *P*(*v*) is the probability density of multi-features vector *O*_*i*_ = (*x*_*i*_, *y*_*i*_, *z*_*i*_, *I*_*i*_, *level of ASM*_*i*_) of *v*. In “The similarity distance based on adaptive local region probability density” section, *P*(*v*) is estimated by the Parzen window estimation method. In order to reduce the computation cost and save the memory, *ASM*_*i*_ is sampled as level 0 (from 0.0000 to 0.1500), level 1 (from 0.1501 to 0.2300), level 2 (from 0.2301 to 0.3500), level 3 (from 0.3501 to 0.5000), level 4 (from 0.5001 to 0.8000) and level 5 (from 0.8001 to 1.0000).

### The PDF-based Bhattacharyya similarity distance for global energy

In order to obtain more stable segmentation and achieve the global optimal segmenting, the global energy term *E*_*global*_ shown as Eq. (2), is incorporated into the proposed integrated ACM model. *E*_*global*_ is global energy term which maximizes the distance of probability density function in different regions. Bhattacharyya distance is applied to measure the distance of probability density distributions between the foreground and background. Bhattacharyya coefficient [27, 28] is defined as Eq. (12).

12 $$ E_{global} = \int_{\Upomega } {\sqrt {P_{ - } (\tau )P_{ + } (\tau )} d\tau } $$

where *E*_*global*_ can be thought of as a direction cosine between two points on the sphere, so its value domain is [0,1]. This measure varies between 0 and 1, where 0 indicates complete mismatch, and 1 indicates a complete match.

In global energy term $E_{global} = \int_{\Upomega } {\sqrt {P_{ - } (\tau )P_{ + } (\tau )} d\tau }$, $P_{ - } \left( {\tau |\phi \left( {\text{x}} \right)} \right)$ and $P_{ + } \left( {\tau |\phi \left( {\text{x}} \right)} \right)$ are kernel-based estimates of the image features observed over the subdomains Ω_−_ and Ω_+_.The kernel-based estimates are given by Eq. (13).

13 $$ \left\{ \begin{aligned} &P_{ - } (\tau |\phi ({\text{x}})) = \frac{{\int_{\Upomega } {K_{ - } } (\tau - \left\| {O({\text{x}})} \right\|)H( - \phi ({\text{x)}})d{\text{x}}}}{{\int_{\Upomega } {H( - \phi ({\text{x)}})d{\text{x}}} }} \hfill \\& P_{ + } (\tau |\phi ({\text{x}})) = \frac{{\int_{\Upomega } {K_{ + } } (\tau - \left\| {O({\text{x}})} \right\|)H(\phi ({\text{x)}})d{\text{x}}}}{{\int_{\Upomega } {H(\phi ({\text{x)}})d{\text{x}}} }} \hfill \\ \end{aligned} \right. $$

where $\tau \in \,{\text{R}}^{N}$, *K*_−_(*τ*) and *K*_+_(*τ*) are two Gaussian density functions that are normalized to have unit integrals with respect to the feature vector *τ*, viz. $\int_{{R^{N} }} {K_{ - } (\tau ){\text{d}}\tau { = }} \int_{{R^{N} }} {K_{ + } (\tau ){\text{d}}\tau { = 1}}$.

### The proposed model and its numerical implementation

The proposed integrated ACM model is shown as Eq. (2). The zero level set that makes the energy function Eq. (2) minimum is the optimal segmentation result. In the simple case, it is obvious that the boundary of the object $\phi$_0_ is the minimizer of the energy functional. The energy function model as Eq. (2) is solved by using variational level set approach. The numerical implementations of energy function as Eq. (14).

14 $$ \begin{aligned} \frac{\partial \phi (v)}{\partial t} &= \delta_{ \in } \{ udiv\left(g\frac{\nabla \phi (v)}{{\left| {\nabla \phi (v)} \right|}}\right) - \lambda_{1} [u(v)]^{m} \int_{{L_{\tau } }} {\left\| {P(\tau ) - P_{1} (\tau )} \right\|} d\tau \hfill \\ &\quad+ \lambda_{2} [1 - u(v)]^{m} \int_{{L_{\tau } }} {\left\| {P(\tau ) - P_{2} (\tau )} \right\|} d\tau \} \hfill \\&\quad + \frac{1}{2}(A_{ - }^{ - 1} - A_{ + }^{ - 1} )\int_{{L_{\tau } }} {\sqrt {P_{ - } (\tau |\phi (v))P_{ + } (\tau |\phi (v))} d\tau } \hfill \\ &\quad+ \int_{{\Upomega_{z} }} {\frac{1}{{A_{ + } }}K_{ + } (\tau - \left\| {O({\text{x}})} \right\|} )\sqrt {\frac{{P_{ - } (\tau |\phi (v))}}{{P_{ + } (\tau |\phi (v))}}d\tau } \hfill \\ &\quad+ \int_{{\Upomega_{z} }} {\frac{1}{{A_{ - } }}K_{ - } (\tau - \left\| {O({\text{x}})} \right\|} )\sqrt {\frac{{P_{ + } (\tau |\phi (v))}}{{P_{ - } (\tau |\phi (v))}}d\tau } \hfill \\ \end{aligned} $$

where $A_{ - } = \smallint _{\Upomega } H\left( { - \phi \left( x \right)} \right)dx$ and $A_{ + } = \int_{\Upomega } {H\,\,( - \phi (x))dx}$ are the size of the image subdomains Ω_−_ and Ω_+_.

### Implementation of potential pulmonary nodule segmentation based on the proposed integrated ACM model

The implementation algorithm for the proposed integrated ACM model is as follows.

1. The pulmonary parenchyma is segmented by an overall segmentation method combining thresholding and morphology, which is shown in Fig. 8.

**Fig. 8:**
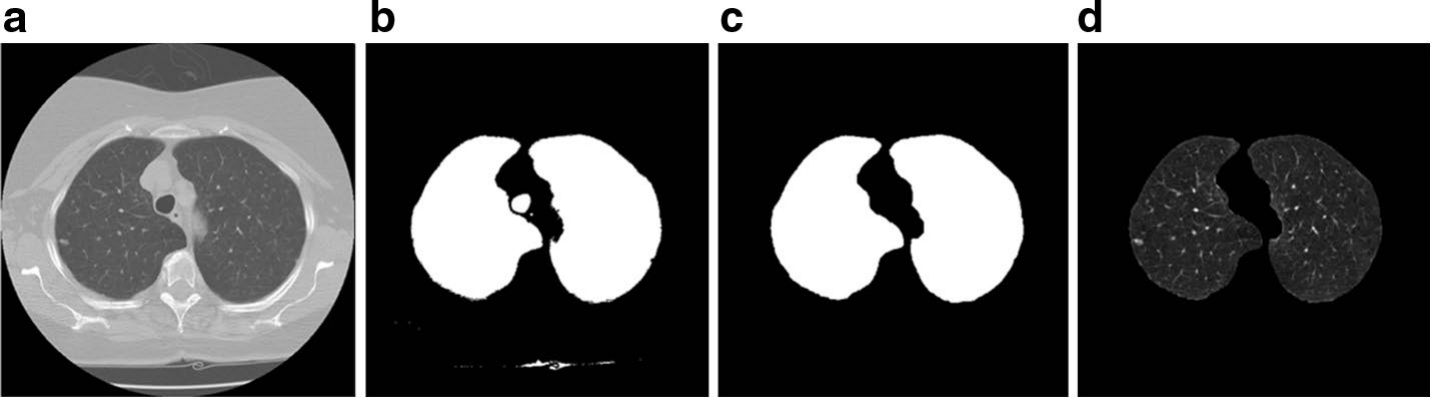
Segmentation results of pulmonary parenchyma. **a** Original image, **b** optimal threshold segmentation, **c** mending image of pulmonary parenchyma based on morphology method; **d** segmentation result of pulmonary parenchyma
2. Multi Feature computation and data structures for data set are constructed.
3. Compute the degree of membership *u*(v). In the proposed model, the fuzzy energy is used as the model motivation power evolving the active contour. As shown in Eq. (2), *u*(*v*): *X* → [0, 1] defines the membership degree of a voxel *v* in data set *D* to the nodule class cluster center. It is different with our previous work [14], the sample in the AFIACM model is *X*_*i*_ = (*x*_*i*_, *y*_*i*_, *z*_*i*_, *I*_*i*_, *ASM*_*i*_). Thus, the degree of membership for each sample *X*_*i*_ = (*x*_*i*_, *y*_*i*_, *z*_*i*_, *I*_*i*_, *ASM*_*i*_) in our model is calculated by using the fuzzy clustering algorithm based on intensity and angular second moment features.
4. Compute the scale parameters *r* and *k* according to “The proposed KNN-based adaptive local energy function” section, and select adaptively local region *R*_*L*_ and *B*(*v*).
5. Specify the stop function term using posterior probability.
6. Implement the numerical algorithm of the proposed model according to Eq. (14). Here *P*(*v*), *P*_1_(*τ*) and *P*_2_(*τ*) in the local region Ω_*τ*_ are computed according to “The similarity distance based on adaptive local region probability density” section and “Generation of multi-dimension feature with high class separability” section. In each iteration, only *P*(*v*) in the local region Ω_*τ*_ near to the curve/surface $\phi$ need to be computed, so pixels/voxels need to be computed are a very small proportion of the total data set. An example is illustrated in Fig. 9. Only *P*(*v*) in the local region Ω_*τ*_ near to the green curve $\phi$ need to be computed. The numbers are 1591, which is 0.6 % of the whole data set (512 × 512).

**Fig. 9:**
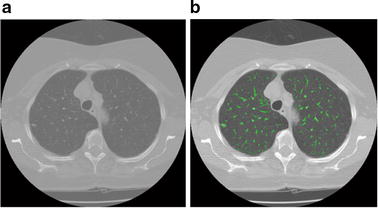
Illustration of pixels/voxels need to be computed, a very small proportion of the total data set. **a** original image, **b** *green curve* $\phi$ in an iteration

## Segmentation refinement of juxta-vascular nodules based on multi-features dynamic clustering

In order to overcome the problem of a small volume over-segmentation in the adhesion region between the juxta-vascular nodule and its attached vessel, and obtain a better segmentation result, a multi-features dynamic clustering method is used for the segmentation refinement of juxta-vascular nodules, which is based on the nodule segmentation using the proposed integrated ACM model and vessel segmentation using FDF-based region growing method. Various types of pulmonary nodules, including GGO nodules (part solid and nonsolid) and juxta-vascular nodules, are segmented under a united segmentation framework. The refinement procession is just used for the pixels/voxels in some boundary regions between the juxta-vascular nodules and blood vessels, without modifying the nodule boundary elsewhere.

### The refinement region selection using the proposed nodule and vessel segmentation method

#### 3D potential vessel segmentation by FDF-based region growing method

In this paper, the 3D vessel segmentation is used as the rough segmentation process of segmentation refinement of juxta-vascular nodules. 3D vessel segmentation can be accomplished by some traditional methods, such as region growing [23] and vessel enhancement filter [29, 30]. However shapes and appearances of vessels are irregular, a satisfactory result often cannot be achieved if only these traditional methods are relied upon. According to the medical prior knowledge, vessels are characterized by a tubular model, the 3D gradient vectors in a vessel can be used to extract a vector in the direction of the vessel by identifying a vector that is approximately orthogonal to the gradients in a local neighborhood [21, 22, 31]. Moreover, an ideal vascular structure not only keeps a certain elongation, but also has no holes. Simply connected domain is a particular connected domain, in which every loop can be continuously pulled to a point without leaving the space [24]. So we can use these shapes and appearances as the constraints of the segmentation method, which are as follows. (1) Since vascular structures should have no holes, the segmented objects must be removed if they are not simply connected. (2) the flow direction can be used as the growing direction constraint condition, which is one of the constraint conditions of region growing method for segmentation of blood vessels and attached nodules.

Here a FDF-based region growing method is proposed for 3D vessel segmentation. In the FDF-based region growing method, the growing constraint conditions include the intensity threshold and flow direction defined by flow direction vector *l*_*d*_. Assume *λ*_1_, *λ*_2_ and *λ*_3_ (*λ*_1_ ≤ *λ*_2_ ≤ *λ*_3_) are eigenvalues of the structure tensor defined by the statistical information of arithmetic average in local region. The structure tensors are computed by directly using the gradient information of each voxel [14, 29]. Let *e*_1_ be the unit length eigenvector belonging to the eigenvalue *λ*_1_. The flow direction vector *l*_*d*_ of the vessel is set as Eq. (15).

15 $$ l_{d} = \sqrt {\lambda_{2} + \lambda_{3} } \cdot e_{1} $$

#### Refinement region selection

In this paper, the intersection between potential vessel region and potential nodules is selected as the refinement region of juxta-vascular nodules based on multi-features dynamic clustering. The processes are as follows.

1. The rough 3D potential vessel segmentation result *S*_*c*1_ is gotten by using the flow direction feature- based region growing method which is described in “3D potential vessel segmentation by FDF-based region growing method” section.
2. The segmentation result *S*_*c*2_ is gotten by using the segmentation method based on the proposed integrated ACM model described in “The proposed integrated ACM with adaptive local region energy and PDF-based similarity distance” section.
3. The potential object *S*_*r*_ for segmentation refinement is ${S_r} = {S_{{c_1}}} \cap {S_{{c_2}}}$.

### Generation and construction of multi-features vector in clustering space

Our proposed multi-features vector takes both the appearance and geometric information into consideration. Because CT values of pulmonary nodules and that of blood vessels are almost uniform, it is difficult to segment exactly the blood vessels and the attached nodules if merely the intensity feature is utilized. So the constructed feature space takes both the appearance and geometric multi-features information into consideration. We build a multi-features space for dynamic clustering in segmentation refinement region. An extended observation vector *X*_*i*_ in the clustering space is defined as Eq. (16).

16 $$ X_{i} = (x_{i} ,\,y_{i} ,\,z_{i} ,\,I_{i} ,\,SI_{i} ) $$

where *x*_*i*_, *y*_*i*_ and *z*_*i*_ are the position features; *I*_*i*_ is the intensity feature; *SI*_*i*_ is the volumetric shape index, reflecting the geometric shape, which is a measure of local shape characteristics [5].

### Implementation of segmentation refinement based on multi-features dynamic clustering

The algorithm for segmentation refinement based on multi-features dynamic clustering is carried out as the following steps.

1. The segmentation refinement region *S*_*r*_ is selected according to “The refinement region selection using the proposed nodule and vessel segmentation method” section.
2. The feature vector *X*_*i*_ = (*x*_*i*_, *y*_*i*_, *z*_*i*_, *I*_*i*_, *SI*_*i*_) is constructed and computed for the points in the region *S*_*r*_, according to “Generation and construction of multi-features vector in clustering space” section.
3. The refinement region *S*_*r*_ is segmented by dynamic clustering method (K-Means clustering method [32]), then the juxta-vascular nodules is segmented exactly.

## Experimental results

In this paper, a clinical dataset of 113 chest CT scans was used to evaluate the proposed method, which consisted of 60 thoracic CT scans obtained from LIDC databases [33] and 53 thoracic CT scans obtained from several big hospitals in Guangzhou City and Shenzhen City, Guangdong province, China. The list of these 60 CT scans from LIDC-IDRI databases is provided in Table 2. The used medical CT slices were data sets with an intensity value of 16bits and a resolution of 512 × 512. Slice thickness varied from 0.5 to 2.5 mm and the total slice number for each scan varied from 49 to 397 with an average of 136/scan. The X-ray tube current ranged from 30 to 280 mA, and the pixel size ranged from 0.5–0.75 mm/pixel. Pulmonary nodules in CT images are solid or GGO (part solid or nonsolid), whose sizes are from 3 to 30 mm. Locations of nodules are uncertain, some are isolated (Solitary Pulmonary Nodule, SPN), others are adhered to blood vessels or lung wall. Each scan was read individually by members of a qualified panel and then a consensual gold standard was defined by the panel. The panel members assigned each nodule to be either GGO, juxta-vascular, GGO juxta-vascular, or others. These nodules are manually segmented. This process defined ground truth of 157 nodules, and the number of different kinds of nodules is shown as Table 3.

**Table 2:** The list of 60 CT scans from LIDC-IDRI databases

	CT scans	Nodule	Type		CT scans	Nodule	Type
1	LIDC-IDRI-0003	Nodule 1	GGO	27	LIDC-IDRI-0160	Nodule 1	JV
		Nodule 2	GGO-JV			Nodule 2	Others
		Nodule 3	Others			Nodule 3	Others 10
		Nodule 4	GGO			Nodule 4	JV
2	LIDC-IDRI-0007	Nodule 1	Others	28	LIDC-IDRI-0162	Nodule 1	Others
3	LIDC-IDRI-0008	Nodule 1	GGO-JV			Nodule 2	JV
		Nodule 2	GGO			Nodule 3	JV
		Nodule 3	Others			Nodule 4	Others 6
4	LIDC-IDRI-0011	None				Nodule 5	Others 7
5	LIDC-IDRI-0015	Nodule 1	Others	29	LIDC-IDRI-0167	Nodule 1	Others
6	LIDC-IDRI-0017	Nodule 1	JV	30	LIDC-IDRI-0168	Nodule 1	JV
7	LIDC-IDRI-0018	None				Nodule 2	Others 12
8	LIDC-IDRI-0019	None		31	LIDC-IDRI-0173	Nodule 1	Others
9	LIDC-IDRI-0021	Nodule 1	GGO	32	LIDC-IDRI-0175	Nodule 1	JV
		Nodule 2	JV	33	LIDC-IDRI-0177	Nodule 1	JV
10	LIDC-IDRI-0025	None		34	LIDC-IDRI-0252	Nodule 1	JV
11	LIDC-IDRI-0032	Nodule 1	Others 2			Nodule 2	GGO-JVvascul
12	LIDC-IDRI-0037	Nodule 1	GGO	35	LIDC-IDRI-0273	nodule 1	JV
		Nodule 2	GGO	36	LIDC-IDRI-0350	Nodule 1	Others 14
		Nodule 3	Others	37	LIDC-IDRI-0477	Nodule 1	JV
13	LIDC-IDRI-0044	Nodule 1	GGO			Nodule 2	JV
		Nodule 2	JV	38	LIDC-IDRI-0580	Nodule 1	JV
		Nodule 3	JV	39	LIDC-IDRI-0626	Nodule 1	GGO
		Nodule 4	GGO	40	LIDC-IDRI-0645	Nodule 1	Others
		Nodule 5	Others	41	LIDC-IDRI-0652	Nodule 1	Others
		Nodule 6	GGO	42	LIDC-IDRI-0681	Nodule 1	GGO
14	LIDC-IDRI-0046	None		43	LIDC-IDRI-0684	Nodule 1	Others
15	LIDC-IDRI-0047	Nodule 1	GGO	44	LIDC-IDRI-0703	Nodule 1	Others
		Nodule 2	JV	45	LIDC-IDRI-0723	Nodule 1	Others
16	LIDC-IDRI-0050	Nodule 1	GGO	46	LIDC-IDRI-0796	Nodule 1	GGO
17	LIDC-IDRI-0051	None		47	LIDC-IDRI-0803	Nodule 1	GGO
18	LIDC-IDRI-0052	Nodule 1	GGO	48	LIDC-IDRI-0818	Nodule 1	Others
		Nodule 2	Others	49	LIDC-IDRI-0828	Nodule 1	GGO
19	LIDC-IDRI-0082	Nodule 1	Others 5	50	LIDC-IDRI-0840	nodule 1	Others
20	LIDC-IDRI-0114	Nodule 1	JV	51	LIDC-IDRI-0865	Nodule 1	GGO
21	LIDC-IDRI-0131	Nodule 1	GGO-JV	52	LIDC-IDRI-0882	Nodule 1	Others
		Nodule 2	JV	53	LIDC-IDRI-0914	Nodule 1	Others
22	LIDC-IDRI-0133	Nodule 1	Others	54	LIDC-IDRI-0928	Nodule 1	GGO
23	LIDC-IDRI-0141	Nodule 1	Others 7			Nodule 2	JV
		Nodule 2	GGO	55	LIDC-IDRI-0938	Nodule 1	GGO
		Nodule 3	Others	56	LIDC-IDRI-0986	Nodule 1	JV
		Nodule 4	JV	57	LIDC-IDRI-0915	Nodule 1	JV
		Nodule 5	GGO	58	LIDC-IDRI-0941	Nodule 1	JV
		Nodule 6	JV			Nodule 2	JV
24	LIDC-IDRI-0146	Nodule 1	JV			Nodule 3	JV
25	LIDC-IDRI-0152	Nodule 1	JV	59	LIDC-IDRI-0953	Nodule 1	JV
26	LIDC-IDRI-0159	Nodule 1	JV			Nodule 2	JV
				60	LIDC-IDRI-1012	Nodule 1	GGO

*JV* juxta-pleural nodule; *GGO*ground-glass opacity nodule; *GGO-JV*GGO juxta-vascular nodule

**Table 3:** The number of GGO, juxta-vascular, GGO juxta-vascular and other nodules

Nodule type	GGO pulmonary nodule	Juxta-vascular pulmonary nodule	GGO Juxta-vascular pulmonary nodule	Others	Total
Number	42	73	7	35	157

“GGO nodule” and “Juxta-vascular nodule” do not include GGO Juxta-vascular pulmonary nodule in the table

Moreover, The different nodule size groups for the GGO nodules, juxta-vascular nodules, GGO juxta-vascular nodules and others is illustrated in Table 4. In our experiment, there are four types of nodules, and they are registered respectively. The performance of our segmentation fusion algorithm was evaluated using both quantitative and qualitative methods. Experimental results in each research step as well as some discussions are presented below.

**Table 4:** The different nodule sizes on testing data

Nodule type	≤ 5 mm	5–10 mm	10–20 mm	Total
GGO pulmonary nodule	7	18	17	42
Juxta-vascular pulmonary nodule	5	49	19	73
GGO Juxta-vascular pulmonary nodule	1	3	3	7
Others	7	15	13	35

“GGO nodule” and “Juxta-vascular nodule” do not include GGO Juxta-vascular pulmonary nodule in the table

### Qualitative validation

#### Validation of the proposed integrated ACM model

**Fig. 10:**
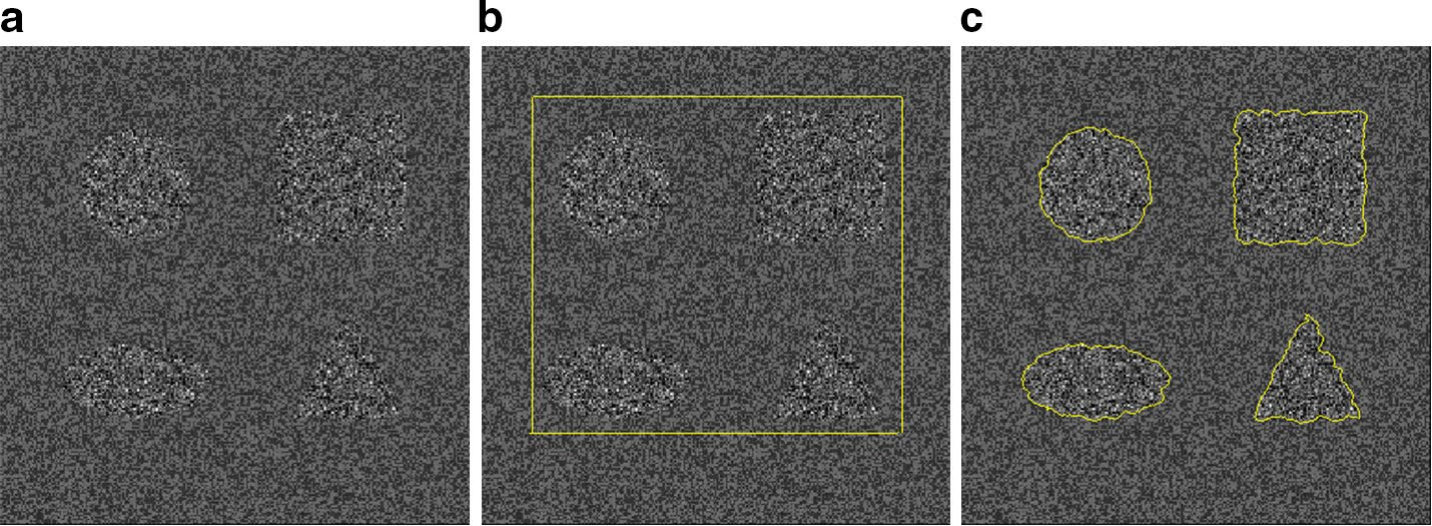
Segmentation of the synthetic image. **a** The original image; **b** the initial contour; **c** the final segmentation

In order to validate the effect of the proposed segmentation method, the synthetic image is used to segmented. As shown in Fig. 10, the problem of boundary leakage at boundaries of objects with intensity inhomogeneity and typical weak edges, whose intensity feature is similar to that of GGO nodules, is solved by using the proposed segmentation method.

#### Validation of 3D potential vessel segmentation by FDF-based region growing method

**Fig. 11:**
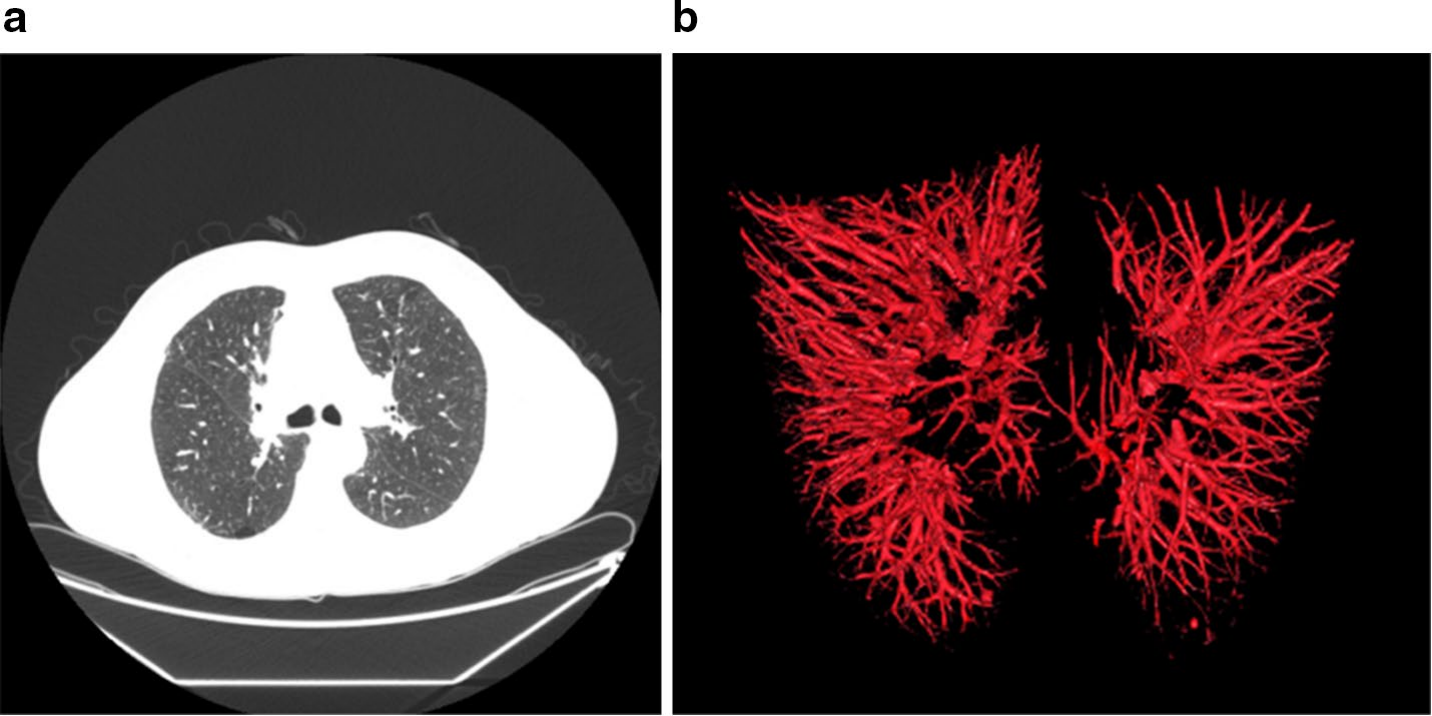
3D potential vessel segmentation result by flow direction feature- based region growing method. **a** one original image (one of No. LIDC-IDRI-0010); **b** the segmentation result

In order to validate the effect of the proposed flow direction feature -based region growing method for 3D potential vessel segmentation, No. LIDC-IDRI-0010 (from LIDC database [33]) is used to segmented. As shown in Fig. 11, 3D potential vessels are segmented successfully by the proposed flow direction feature- based region growing method. It is validated that the method can be used as the segmentation of 3D potential vessels and rough segmentation of juxta-vascular pulmonary nodules.

#### Validation of multi-features dynamic clustering method

**Fig. 12:**
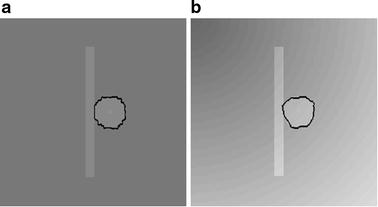
Segmentation of the synthetic image. **a** The segmentation result by the proposed multi-features dynamic clustering segmentation method, in clear background; **b** the segmentation result by the proposed multi-features dynamic clustering segmentation method, in background with intensity inhomogeneity

In order to validate the effect of the proposed multi-features dynamic clustering segmentation method, the synthetic image is used to segment. As shown in Fig. 12, the tubular model and round or oval-shaped model can be segmented successfully. This implies the multi-features dynamic clustering method can be used for segmentation of juxta-vascular nodules, since vessels can be characterized by the tubular models and a pulmonary nodule is a small round or oval-shaped growth in the lung.

#### Validation of the proposed segmentation method of nodules

In order to validate the effect of the proposed segmentation method, the clinical data with GGO nodules and juxta-vascular nodules should be segmented and explored.

**Fig. 13:**
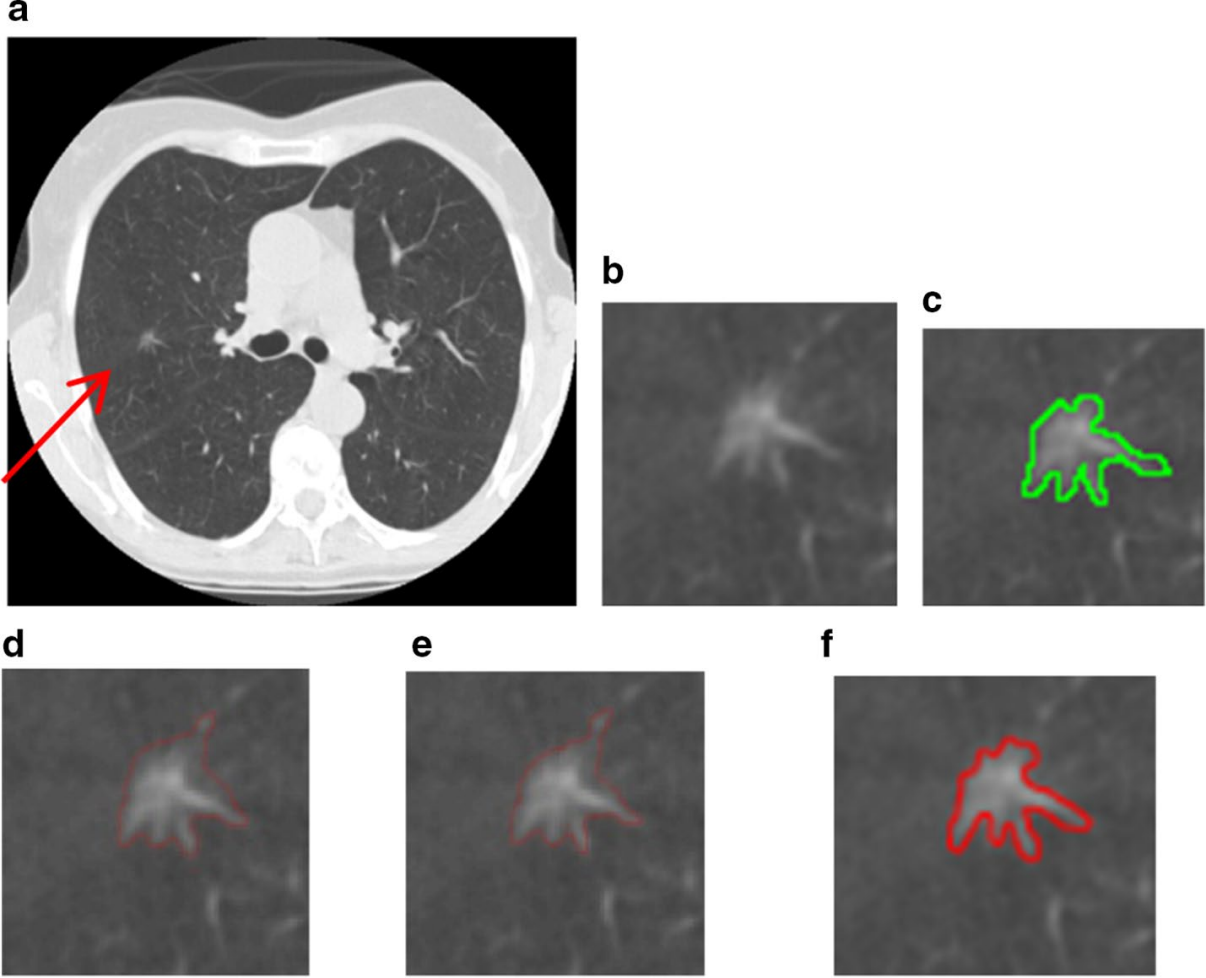
Segmentation results of the GGO pulmonary nodule. **a** original CT image; **b** local zooming of Fig. 13a; **c** the delineation result by an experienced radiologist; **d** the segmentation result by region-based active contour model; **e** the segmentation result by traditional integrated active contour model; **f** the segmentation result by the proposed segmentation method

A comparison for segmentation of GGO nodule between the proposed segmentation method and the traditional approach [34], e.g. region-based active contour model [6, 7] and integrated active contour model [13], is shown in Fig. 13. As shown in Fig. 13d, e, the problem of boundary leakage at boundaries of a GGO pulmonary nodule occurs, while the problem is solved in Fig. 13f.

**Fig. 14:**
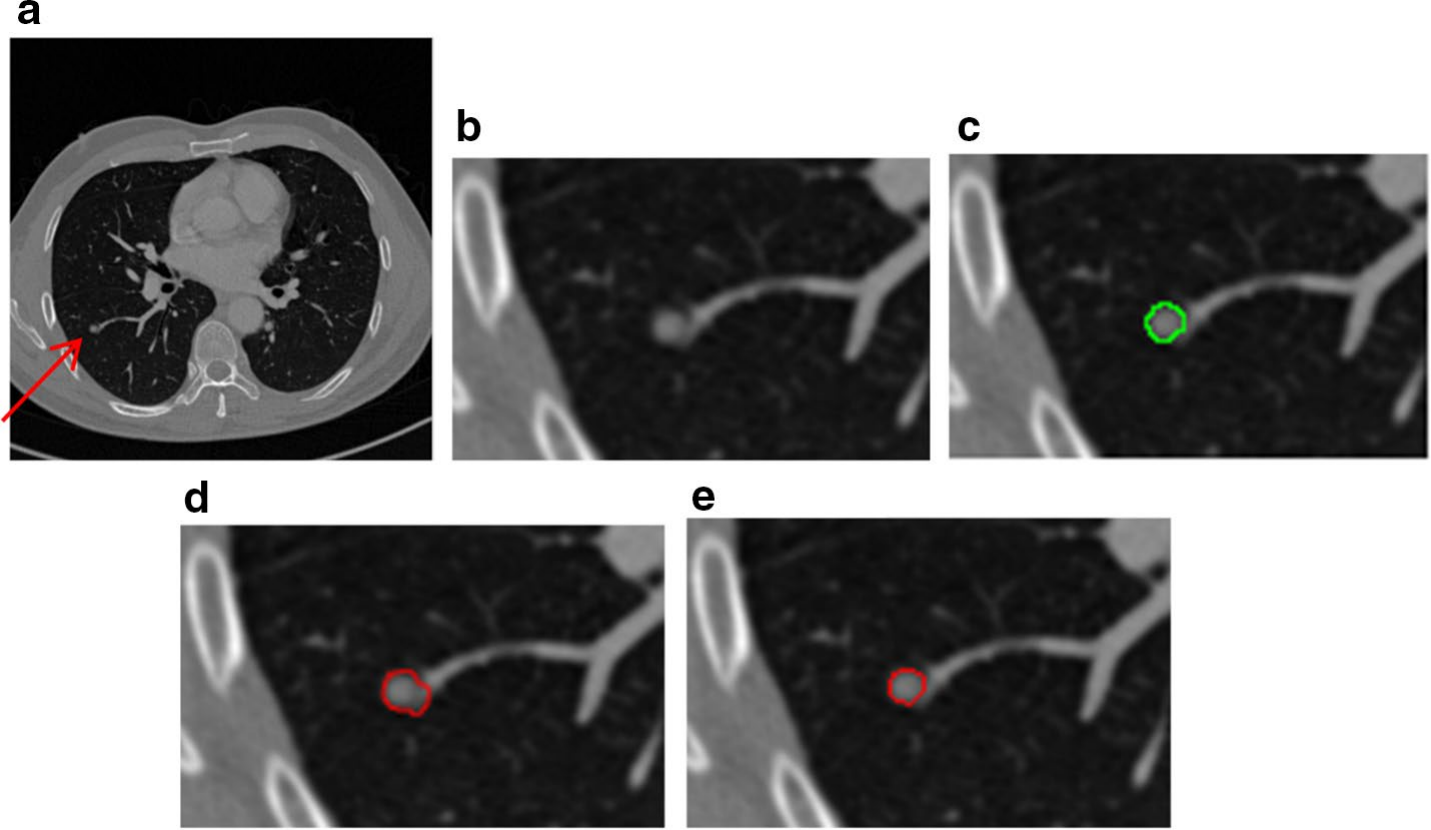
Segmentation results of the juxta-vascular pulmonary nodule. **a** Original CT image; **b** local zooming of Fig. 14a; **c** the delineation result by an experienced radiologist; **d** the segmentation result by the segmentation method without using the proposed refinement method; **e** the segmentation refinement result by the proposed multi-features dynamic clustering

**Fig. 15:**
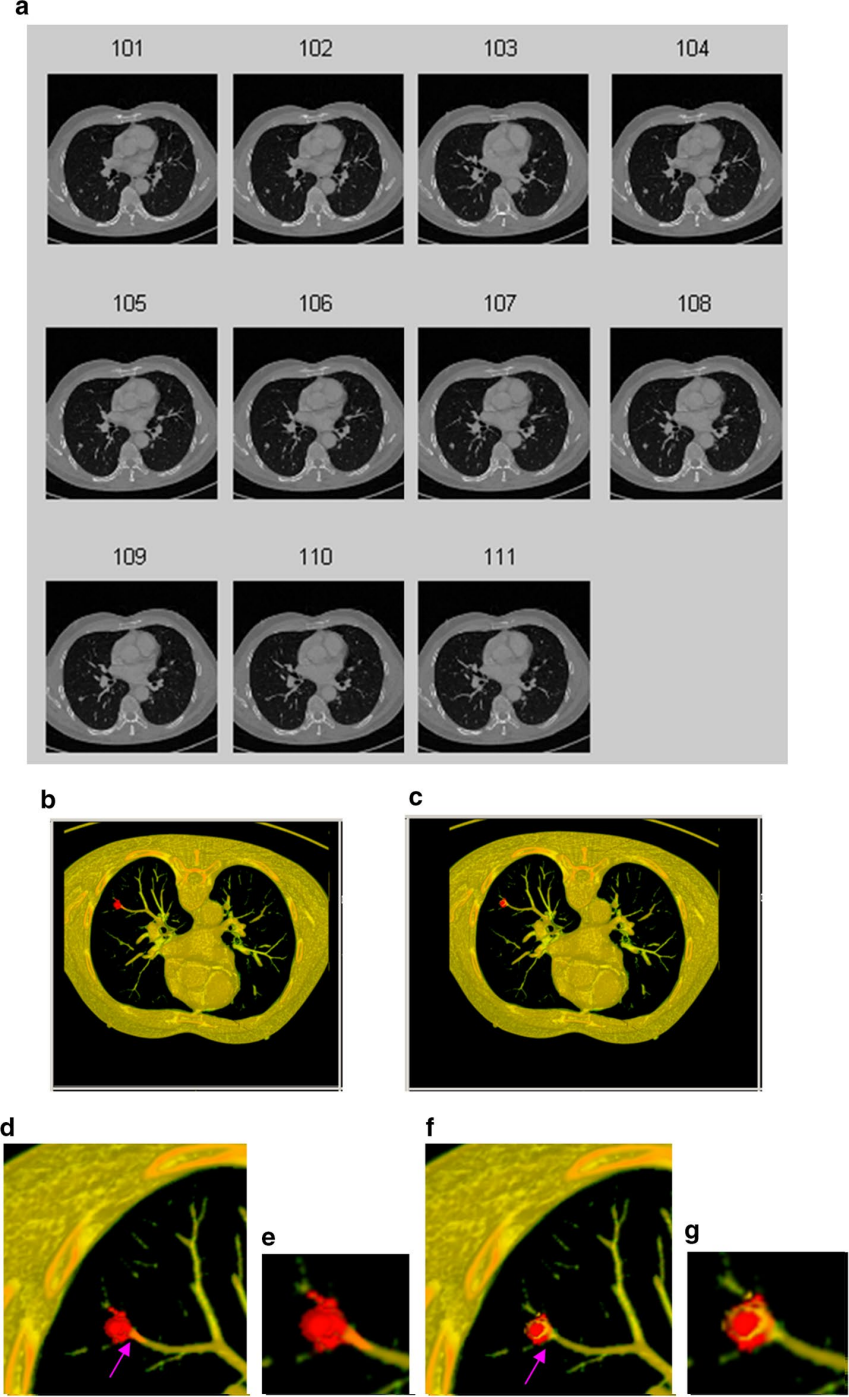
3D segmentation results of the juxta-vascular pulmonary nodule. **a** original CT image sequence; **b** the 3D segmentation result without using the proposed refinement method; **c** the 3D segmentation result using the proposed segmentation method with multi-features dynamic clustering refinement; **d** local zooming of Fig. 15b; **e** local zooming of Fig. 15d; **f** local zooming of Fig. 15c; **g** local zooming of Fig. 15f

In this paper, we handle various types of nodules under a united framework. Firstly the image sequences are segmented by using the proposed segmentation method. Then the juxta-vascular nodules are segmented finely by using segmentation refinement based multi-features dynamic clustering method. Some experimental results for segmentation of juxta-vascular pulmonary nodule are shown in Figs. 14 and 15. From Fig. 15, we can see that the refinement procession is only applied to voxels in some regions *S*_*r*_ containing the blood vessels and juxta-vascular nodules. The refinement method will not modify the nodule boundary elsewhere, so the problem of over-segmentation does not occur. It is validated that the method can be used as the segmentation of juxta-vascular pulmonary nodules.

**Fig. 16:**
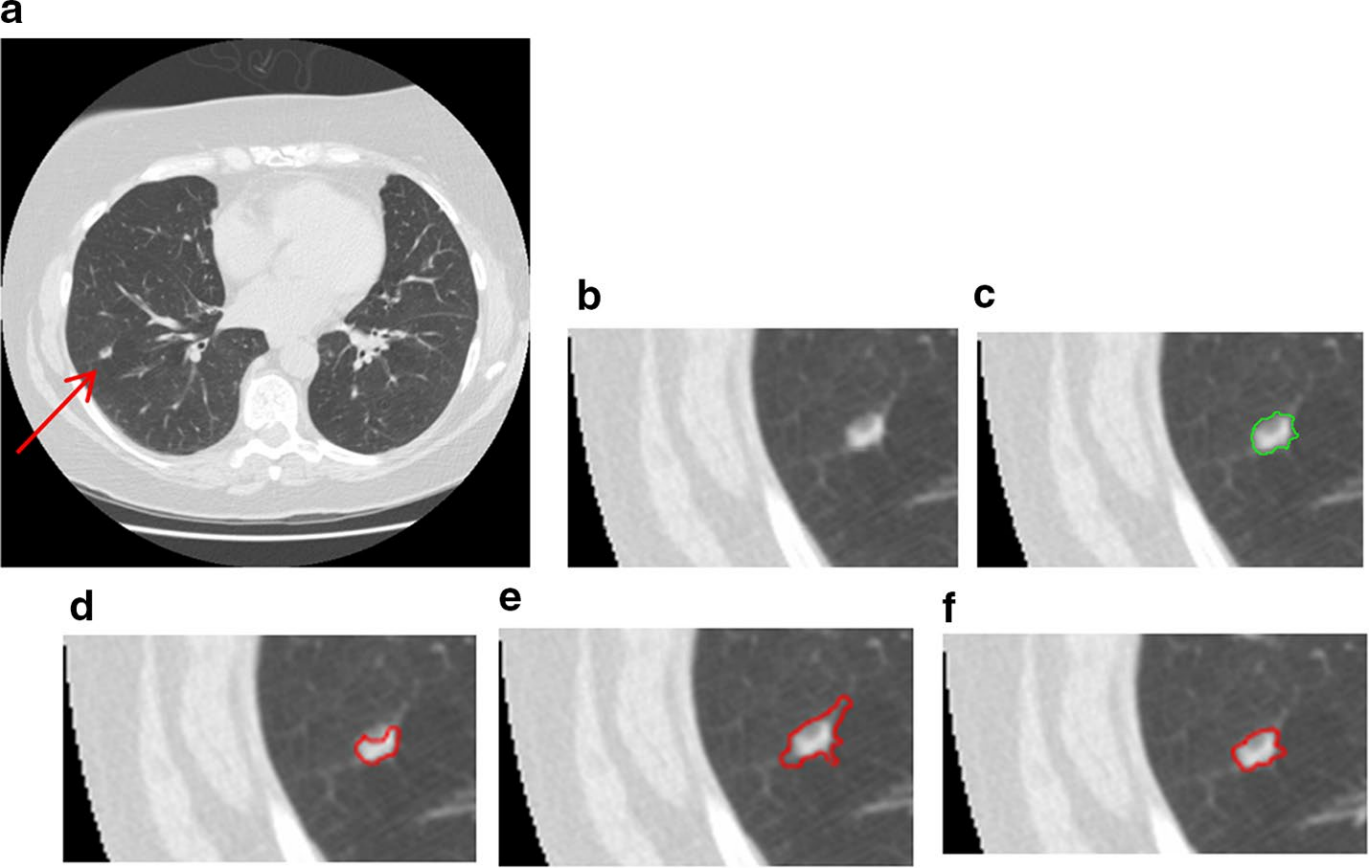
Segmentation result of a GGO juxta-vascular pulmonary nodule. **a** Original CT image; **b** local zooming of Fig. 16a; **c** the delineation result by an experienced radiologist; **d** the segmentation result by region-based active contour model; **e** the segmentation result by traditional integrated active contour model; **f** the segmentation result by the proposed method

Figure 16 shows the segmentation results of a GGO juxta-vascular pulmonary nodule using the proposed method, region-based ACM [6, 7] and the integrated ACM [13, 34], respectively. As shown in Fig. 16d, e, the problem of boundary leakage and over segmentation occurs in the adhesion place between the juxta-vascular nodule and its attached vessel; while as shown in Fig. 16f, the problem of boundary leakage and over-segmentation is solved by the proposed segmentation method.

From Figs. 13, 14, 15 and 16, the described segmentation method outperforms the traditional methods.

**Fig. 17:**
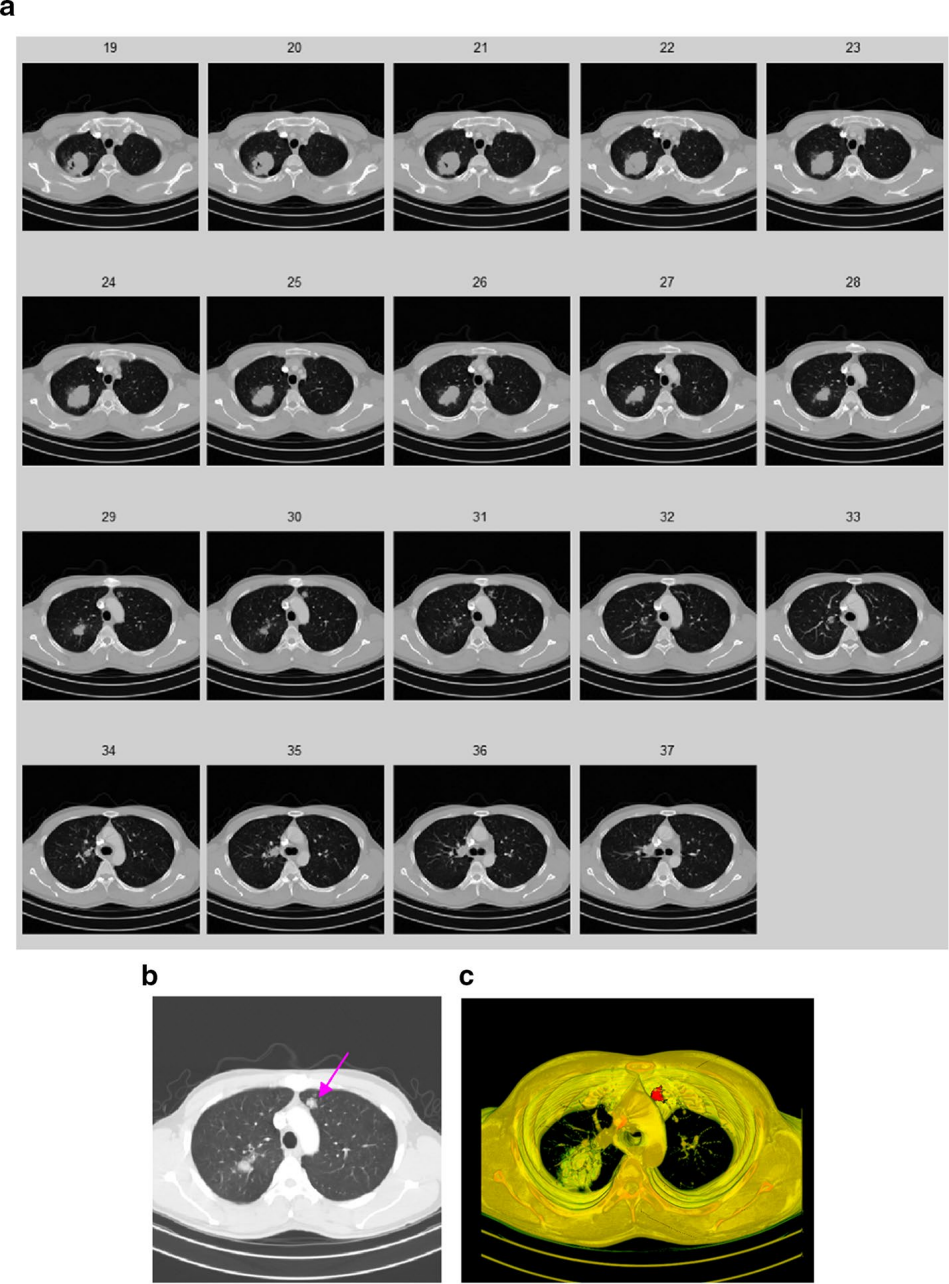
3D segmentation results of the GGO pulmonary nodule. **a** Original CT image sequence; **b** the GGO nodule; **c** the 3D segmentation result by using the proposed method

**Fig. 18:**
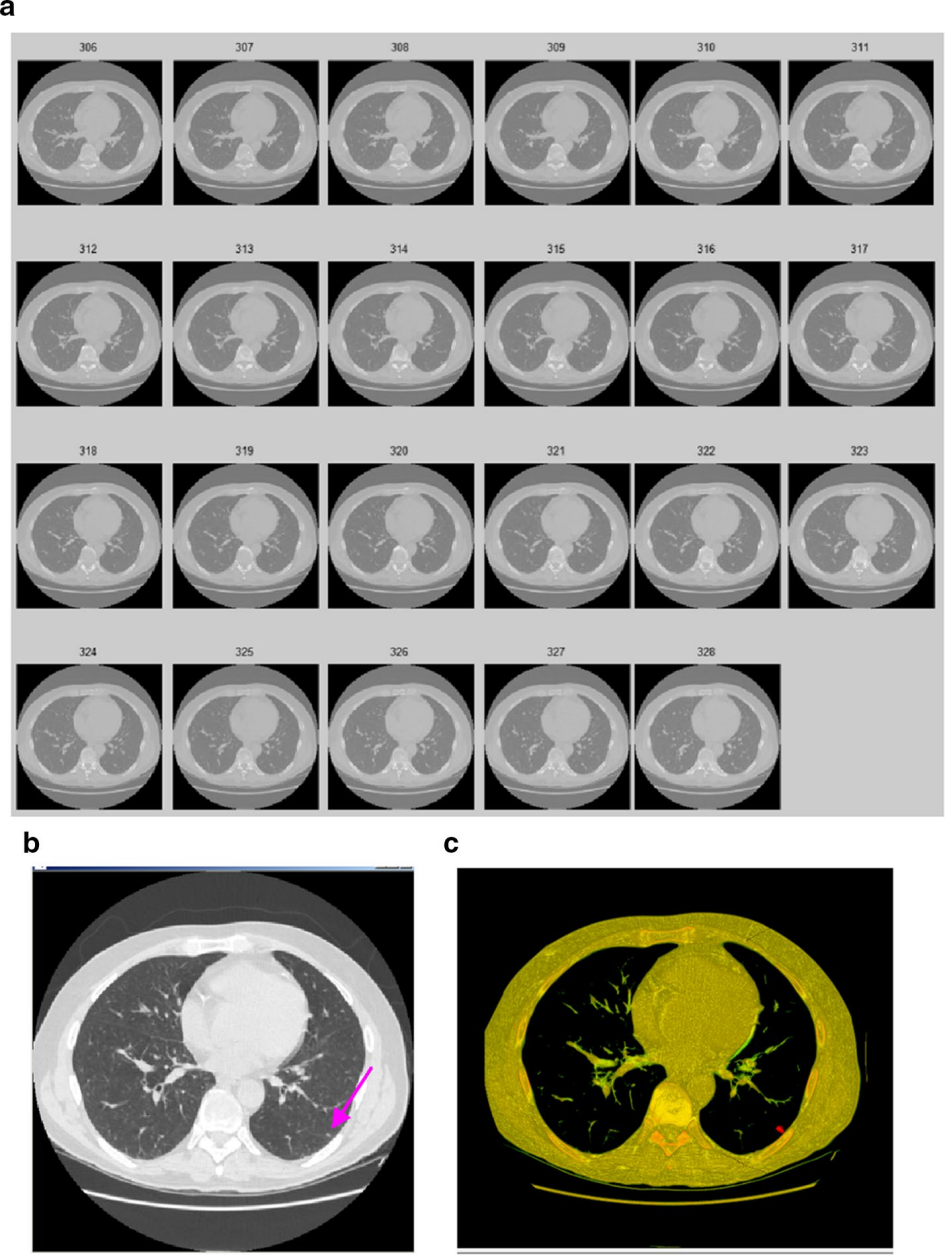
3D segmentation results of the GGO pulmonary nodule. **a** Original CT image sequence; **b** the juxta-pleural nodule; **c** the 3D segmentation result by using the proposed method

Other classical experimental results for segmentation of juxta-vascular pulmonary nodule are is shown in Figs. 17 and 18. It is validated that the method can be used as the segmentation of various types of pulmonary nodules.

### Quantitative validation

Beyond the visual inspection, a quantitative analysis is necessary to ascertain the accuracy of the proposed segmentation method. Here, the well known Tanimoto/Jaccard error *A*(*C*_*m*_, *C*_*o*_) as Eq. (5) is used as the validation merics, which refers to distances between segmentation results or to volume overlaps between the gold standard and the proposed segmentation method. The gold standard typically is a high-quality reference segmentation carried out by experts.

Segmentation measure results are shown in Table 5. Table 5 shows that the errors of the proposed method are less than the other three traditional methods, the edge-based active contour model, the traditional region-based active contour model and the integrated active contour model [6, 7, 13, 34].

**Table 5:** Segmentation measure results (error rate)

CT image	The edge-based active contour model	The region- based active contour model	The traditional integrated active contour model	The proposed active contour model
GGO pulmonary nodule	Mean 0.31 Std. 0.11	Mean 0.36 Std. 0.13	Mean 0.36 Std. 0.09	Mean 0.17 Std. 0.07
Juxta-vascular pulmonary nodule	Mean 0.32 Std. 0.13	Mean 0.39 Std. 0.12	Mean 0.29 Std. 0.12	Mean 0.20 Std. 0.09
GGO Juxta-vascular nodule	Mean 0.43 Std. 0.12	Mean 0.47 Std. 0.09	Mean 0.41 Std. 0.13	Mean 0.24 Std. 0.08

Standard deviation (Std.)

“GGO nodule” and “Juxta-vascular nodule” do not include GGO Juxta- vascular pulmonary nodule in the table

In our experiment, there are 3 nodules missed in the experimental test set of 157 nodules in total. Two of them are small GGO nodules close to 3 mm, and one of missing nodules is a small juxta-pleural nodule with very low contrast and close to 3 mm.

The proposed segmentation algorithm was implemented and tested on the computer with 3.46 × 2 GHz CPU, 192 GB Memory and Graphic Card (GPU memory 12 GB GDDRS, 317 GB/s). On average, it takes about 3.14 min/scan (about 1.37 s/each image), which does not include the cost for data pre-processing.

## Discussion

Experimental results of segmentation for pulmonary nodules show desirable performances of the proposed segmentation method using the test dataset. The segmentation performance for GGO, juxta-vascular and GGO juxta-vascular nodules was an average Tanimoto/Jaccard error of 0.17, 0.20 and 0.24, respectively.

We attempt a comparison with the results reported by other research groups. Kubota et al. [1] proposed a segmentation method based on morphological approaches and convexity models for segmenting the pulmonary nodules of various densities. Results on 21 LIDC cases were reported with segmentation overlap measures of mean 0.69 and standard deviation 0.18. Diciotti et al. [2]. presented a refinement method for the segmentation of juxta-vascular nodules, which was based on a local shape analysis of the initial segmentation making use of geodesic distance map representations. They observed a percentage of successful segmentations of 84.8 % in fully automated mode and of 91.0 % by using an additional interactive mode for improving the segmentation quality of juxta-vascular nodules. However, GGO juxta-vascular nodules were not reported in their work. Kostis et al. [21] collected 21 juxta-vascular nodules, and observed an 80 % successful rate. Okadaetal et al. [35] reported an 81.2 % estimation rate on a dataset of 1310 various types of nodules (3–30 mm in diameter). Unfortunately, though most of algorithms have been developed for lung nodules, most authors did not report quantitative results for various types of nodules. Ye et al. [5] proposed a shape-based SVM method for detecting nodules. The 3-D local geometric and statistical intensity features were used to detect potential solid and GGO nodule. But the segmentation results were not been reported. Murphy [32] used the local image features of shape index and curvedness to detect candidate structures in the lung volume, but the segmentation results were not been reported yet.

Comparing with different segmentation methods covered in literature [3] and other reported literatures above, it seems that the proposed method’s relatively precise segmentation performances. The reasons why the proposed method has a better performance for segmenting all types of GGO and juxta-vascular nodules are as follows. In the proposed integrated ACM model, the local domain for local energy model is selected adaptively, and measurable distances between probability density functions of multi-dimension features with high class separability are used. The model using local information commonly can obtain better performance than that of global statistic information in solving the segmentation problems of intensity inhomogeneity, such as part solid and nonsolid GGO nodules. Multi-dimension features are also important and helpful for the segmentation of GGO nodules. In many research papers, juxta-vascular nodules observed in CT images are outlined applying a global refinement procedure (i.e., throughout the initial segmentation boundary) after an initial rough segmentation. Differently, as is mentioned before, our solution for efficiently segmenting the potential nodule objects involves two steps: (1) a segmentation method is proposed for a whole segmentation, which is based on the proposed integrated ACM model. The method uses an adaptive local region energy model with PDF-based similarity distance, which is especially used for low-contrast nodules such as part solid and nonsolid GGO nodules, to overcome the problems of boundary leakage, intensity inhomogeneity; (2) a segmentation refinement method based on multi-features dynamic clustering method, which is referred to as a fine segmentation, is used to segment potential juxta-vascular nodules. So the correction method has the advantage that it locally refines the nodule segmentation along recognized vessel attachments only, without modifying the nodule boundary elsewhere.

However, some nodules are missed by the proposed segmentation method. Typically, these nodules are too small (almost 3 mm), or small juxta-pleural nodules with very low contrast, which makes it difficult to segment. The small GGO juxta-pleural nodule with pleural tail is very near to the edge of lung wall.

To further improve the segmentation performance, some improvements need to be further investigated as follows: in order to recognize small and juxta-pleural pulmonary nodules in noisy image more effectively, an adaptive smoothing method needs to be further investigated, and the juxta-pleural nodules should be further researched; This requires further investigated in more detail.

## Conclusions

When the traditional segmentation method is used to segment the GGO and juxta-vascular nodules with weak edges and intensity inhomogeneity characteristic, the problems of boundary leakage and small volume over-segmentation often appear. To solve these problems, a novel segmentation method is proposed for pulmonary nodules in CT images, which is based on the proposed integrated ACM model and multi-features dynamic clustering method, especially for GGO nodules (part solid and nonsolid) and juxta-vascular nodules. This study demonstrates the superiority of the proposed method. The described segmentation method outperforms the traditional methods, and evaluating the algorithm on the provided test data leads to an average Tanimoto/Jaccard error of 0.17, 0.20 and 0.24 for GGO, juxta-vascular and GGO juxta-vascular nodules, respectively.
